# Vibration Control of AFG Beam with Moving Load in Thermal Environment

**DOI:** 10.3390/ma18030725

**Published:** 2025-02-06

**Authors:** Xi Xu, Yuewu Wang

**Affiliations:** Department of Mechanics, Beijing University of Technology, Beijing 100124, China; xux991103@emails.bjut.edu.cn

**Keywords:** AFG, moving load, nonlinear energy sink (NES), parameter optimization

## Abstract

Forced vibrations resulting from moving loads, along with efficient vibration control, are essential in transportation engineering, earthquake engineering, and aerospace engineering. In this study, the vibrational response of an axially functionally graded (AFG) beam subjected to a moving harmonic load within a thermal environment was investigated. The primary aim was to explore the potential of controlling this vibration by incorporating a nonlinear energy sink (NES). A model for the AFG beam, with clamped–clamped boundary conditions, was developed using Euler–Bernoulli beam theory and the Lagrange method, accounting for the effects of the thermal environment and the moving load. The numerical simulations were performed using the Newmark method to solve the governing equations. The results demonstrated the effectiveness of the NES in mitigating the vibrational response of the beam under thermal and dynamic loading conditions. The effective reduction of maximum deflection caused by moving loads was set as the optimization objective to identify the most optimal parameters of the NES. The results were presented through a series of parameter analyses, revealing that the nonlinear damper can quickly dissipate the beam’s energy when the loads exit the structure. Furthermore, a properly designed NES can result in a 2.4-fold increase in suppression efficiency.

## 1. Introduction

The concept of functionally graded materials (FGMs) was introduced in 1984 by materials scientists in the Sendai area as a novel type of heat-insulating material [1]. FGMs are composites characterized by a gradual variation in their properties across different directions. Typically, these materials are composed of a combination of ceramics and metals. In recent years, FGMs have found diverse applications across various engineering fields, including aerospace, space vehicles, and the biomedical sector. With their growing use, researchers have increasingly focused on investigating the mechanical behavior of FGMs. For instance, Loy et al. examined the vibration behavior of functionally graded cylindrical shells [2], while Patel et al. applied higher-order theory to investigate the free vibration of functionally graded elliptical cylindrical shells [3]. Wang et al. studied the nonlinear dynamic response of inhomogeneous functional plates [4]. Xue et al. explored the free vibration of functionally graded porous cylindrical panels and shells [5]. The findings indicate that the vibration characteristics of these structural components are significantly enhanced by utilizing composite materials.

On the other hand, FGM structures frequently function under complex conditions, such as high-temperature environments, which can lead to significant changes in the mechanical properties of functionally graded (FG) beams. As a result, the vibration behavior of FGM structures in thermal environments has become a critical research topic and remains a focal point for scientific inquiry. The following are some previous works. Huang et al. studied the nonlinear vibration and dynamic response of an FGM plate with surface-bonded piezoelectric layers in a thermal environment [6]. Jagtap et al. discussed the stochastic nonlinear free vibration response of an elastically supported FGM plate resting on a two-parameter Pasternak foundation [7]. Tung et al. presented an analytical approach to study the nonlinear stability of clamped FGM shallow spherical shells and circular plates resting on elastic foundations, subjected to uniform external pressure, and exposed to thermal environments [8]. Yadav et al. analyzed the nonlinear vibration response of FG circular cylindrical shells subjected to a thermal environment with a mechanical in-plane nonuniformly-distributed loading along the edges and harmonic radial force [9]. Wang et al. investigated the free vibration of a rotating FG spherical–cylindrical–conical shell with arbitrary boundary conditions in a thermal environment based on the first-order shear deformation theory for shells [10]. The above-mentioned references all focus on the free vibration of composite material structures and do not address the forced vibration response. They all found that an increase in temperature leads to a decrease in the structure’s natural frequency. The topic of how temperature affects vibration responses induced by moving loads and the effectiveness of vibration control continues to attract significant attention from researchers for in-depth studies.

As mentioned earlier, moving load analysis is a very important topic in the structural analysis of bridges, roads, railways, etc. Şimşek studied the nonlinear dynamic response of an FGM beam subjected to a moving load using various shear deformation theories [11]. Wayou et al. investigated the nonlinear dynamics of an Euler–Bernoulli beam under moving loads to handle the influence of the load inertia as well as nonlinearity [12]. Wang et al. investigated the free and forced vibrations of an FG porous tube under moving distributed load using the refined beam theory [13]. Zhang et al. first attempted to analyze the nonlinear temperature-dependent dynamic response of a porous FG graphene-nanoplatelet-reinforced composite cylindrical panel under moving load [14]. Most existing studies focus on FG structures graded along the thickness direction, but in certain special cases, grading along the axial direction may be a better choice to achieve specific mechanical properties. The axially functionally graded (AFG) materials are one type of specialized FG structure used in various industries. Hein et al. used Haar wavelets to study the free vibrations of non-uniform and axially functionally graded beams [15]. Şimşek et al. investigated the dynamic behavior of an axially functionally graded beam under a moving harmonic load [16]. Huang et al. studied free vibration of axially functionally graded Timoshenko beams with non-uniform cross-section [17]. Li et al. used nonlocal strain gradient theory to study bending, buckling and vibration of axially functionally graded beams [18]. Although some researchers have studied functionally graded beams graded along the axis, the research on these beams remains limited, with little attention given to vibration reduction in such structures.

In practical applications, such as moving train loads, the vibration amplitude of the bridge is generally too large and thus affects the comfort of the passengers. Many methods to control and mitigate bridge vibration have been proposed [19,20], of which the nonlinear energy sink (NES) is one such method. The concept of NES was proposed in 2000 [21] and developed based on a dynamic vibration absorber. An NES is a lightweight absorber with pure nonlinear stiffness, i.e., whose linear stiffness is close to zero. Due to its cubic stiffness, it can dissipate the energy of the main system in a one-way, irreversible manner to achieve vibration reduction. Owing to its advantages of wide frequency vibration absorption and light weight, it has attracted considerable attention from researchers. They have studied the performance of various NES, such as parallel nonlinear energy sinks [22], lever-type nonlinear energy sink [23], inertial nonlinear energy sink [24] and fractional nonlinear energy sinks [25]. Ding et al. also studied the design, analysis and application of NES [26]. The existing research studies have shown that the vibration absorption efficiency of an NES is higher than that of a traditional vibration absorber [27]. In practical applications, such as moving train loads on bridges, excessive vibration can affect passenger comfort. Various methods have been proposed to mitigate such vibrations. The nonlinear energy sink (NES), a lightweight absorber with pure nonlinear stiffness, has shown superior performance in vibration absorption compared to traditional absorbers. Despite the progress in NES research, only a few studies have applied NES to AFG structures under moving loads in thermal environments.

To fill the research gaps mentioned above, this study makes the first attempts to investigate the vibration control of the AFG beams under moving loads. In this study, the vibration of an AFG beam subjected to a moving harmonic load in a thermal environment was investigated, with vibration control achieved by incorporating an NES. The equations of motion were derived using the Euler–Bernoulli beam theory and the Lagrange method. The transverse deflection of the AFG beam was expressed in modal form, and appropriate NES parameters were selected for vibration control. The dynamic response of the system was analyzed using the Newmark method, and the results with and without NES were compared. The findings showed that the NES effectively reduced the vibration amplitude of the system in a thermal environment. This research contributes to the development of vibration control methods for AFG structures, an area that remains underexplored in the existing literature.

## 2. Modeling and Methodology

### 2.1. Modeling

**Figure 1:**
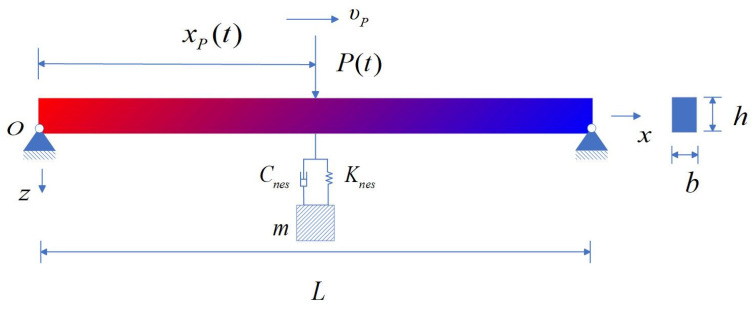
Schematic of an AFG beam with NES subjected to a uniform thermal load.

In this study, an AFG beam with a rectangular cross-section, characterized by its length *L*, width *b*, and height *h*, is considered. The beam is subjected to two external loads: a uniform thermal load, resulting in a temperature increase of Δ*T*, and a moving load, *P*(*t*), which travels at a constant velocity in the *x*-direction. To mitigate the vibrations induced in the composite laminated beam, a Nonlinear Energy Sink (NES) is introduced. The NES consists of mass, damping, and cubic stiffness elements, which are strategically placed to absorb the vibrational energy. A schematic of the AFG beam, along with the NES and the imposed thermal and moving loads, is provided in Figure 1.

**Figure 2:**
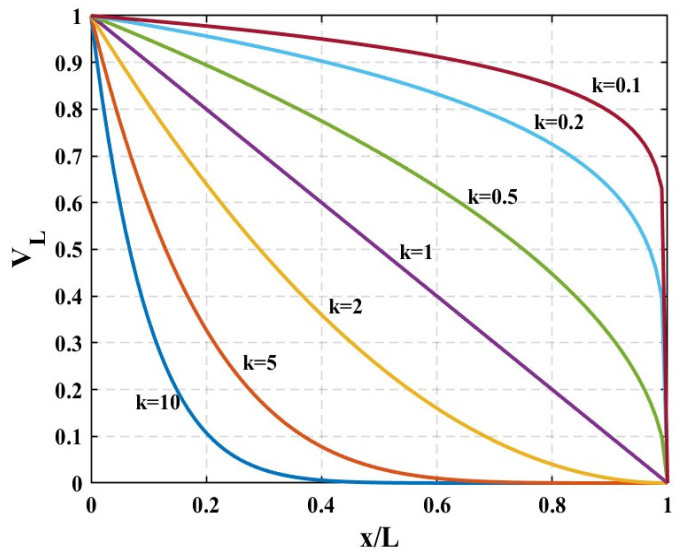
Alteration of the volume fraction of the component at the left end of the AFG beam.

We assume that the volume fraction of the components of the AFG beam varies axially according to a power-law distribution, which can be expressed as follows:

(1) $$V_{L}={\left(1-x/L\right)}^{k}$$

(2) $$V_{L}+V_{R}=1$$

where *V_L_* and *V_R_* are the volume fractions of the left- end right-end components, respectively, and the power-law index is expressed as *k*. Figure 2 displayed the variation of *V_L_* along the length of the beam for different values of *k*.

In this study, the rule of mixtures, specifically the Voigt model, was analyzed to describe the effective properties of functionally graded (FG) materials. The Voigt model, which assumes the material components are aligned in parallel, is relatively straightforward and computationally efficient compared to other more complex models. Despite its simplicity, it provides valuable insights into the macroscopic behavior of AFG beams, particularly in capturing the general trends of how the material properties—such as stiffness and elasticity—vary across the structure. While the Voigt model does not account for more intricate aspects of material distribution or gradients that may be present in real-world applications, it serves as a useful approximation, especially for initial analyses and understanding the influence of varying constituent materials on the overall performance of AFG beams. It is assumed that *V_L_* in both Voigt models follows a non-complicated power-law form, as given by Equation (1).

Under the Voigt model, the effective properties of the beam can be gained using Equation (3)

(3) $$P(x)=P_{L}V_{L}+P_{R}V_{R}=(P_{L}-P_{R}){\left(1-x/L\right)}^{k}+P_{R}$$

where *P_L_* and *P_R_* are the effective material properties (i.e., the modulus of elasticity, *E*, mass density, *ρ*, Poisson’s ratio, *ν*, and the coefficient of thermal expansion, *α*) of the beam at the left and the right end, respectively. *P*(*x*) expresses the effective material properties of the AFG beam.

Therefore, the elastic modulus, density, and coefficient of thermal expansion of the AFG beam are:

(4) $$E(x)=(E_{L}-E_{R}){\left(1-x/L\right)}^{k}+E_{R}$$

(5) $$\rho (x)=(\rho_{L}-\rho_{R}){\left(1-x/L\right)}^{k}+\rho_{R}$$

(6) $$\alpha (x)=(\alpha_{L}-\alpha_{R}){\left(1-x/L\right)}^{k}+\alpha_{R}$$

### 2.2. Methodology

In this paper, the transverse vibration characteristics of an AFG beam were investigated using the Euler–Bernoulli theory. While this theory neglects shear deformation, it is a suitable idealization for slender beams where the length-to-thickness ratio is large. The primary aim of this study was to examine the impact of NES on the dynamic response of AFG beams. To ensure the applicability of the Euler–Bernoulli beam theory, a slender beam with a thickness-to-length ratio of 1/40 was considered, which allows for accurate results under the assumptions of this theory. As illustrated in Figure 1, the deformation of the AFG beam was presumed to be in the *x*-*z* plane. The displacement components in the *x* and *z* directions are denoted as *u_x_* and *u_z_*, respectively. For the CBT, the displacement field can be expressed as follows [28]:

(7) $$\begin{array}{c} u_{x}=-z\frac{\partial w(x,t)}{\partial x}\\ u_{z}=w(x,t) \end{array}$$

where *w*(*x,t*) represent the transverse displacement at an arbitrary point on the neutral axis. The kinematic relationship based on the above displacement field is expressed as:

(8) $$\varepsilon_{xx}=\frac{\partial u_{x}}{\partial x}$$

Using Hooke’s law, the stress of the beam can be obtained as follows:

(9) $$\sigma_{xx}=E(x)\varepsilon_{xx}$$

where *σ_xx_* is the normal stress and *E*(*x*) is Young’s modulus, which is the function of the longitudinal coordinate.

In this study, the AFG beam is subjected to a thermal environment, where the temperature increases uniformly by Δ*T*. Assuming that both ends of the beam are fixed, the in-plane thermal force at each section of the AFG beam can be determined using the following relation:

(10) $$N_{x}^{T}=\varepsilon_{x}^{T}\sigma_{x}^{T}=\Delta TE(x)A\alpha (x)$$

where $\varepsilon_{x}^{T}$ and $\sigma_{x}^{T}$ are the transverse thermal strain and stress, respectively.

(11) $$\varepsilon_{x}^{T}=\alpha (x)\Delta T$$

(12) $$\sigma_{x}^{T}=E(x)\varepsilon_{x}^{T}$$

The strain energy, *U*, and the kinetic energy, *V*, of the beam can be expressed as

(13) $$U=\frac{1}{2}\int_{0}^{L}\int_{A}\sigma_{xx}\varepsilon_{xx}dAdx$$

(14) $$V=\frac{1}{2}\int_{0}^{L}\int_{A}\rho (x)\left[(\frac{\partial u_{x}}{\partial t}{)}^{2}+(\frac{\partial u_{z}}{\partial t}{)}^{2}\right]dAdx$$

The potential energy of the in-plane force, *N_x_^T^*, can be written as

(15) $$U^{T}=-\frac{1}{2}\overset{L}{\underset{0}{\int }}N_{x}^{T}(\frac{\partial w(x,t)}{\partial x}{)}^{2}dx$$

The potential energy of the moving harmonic load can be written as

(16) $$U^{P}=-\overset{L}{\underset{0}{\int }}P(t)\delta (x-\nu_{p}t)w(x,t)dx$$

*P*(*t*) denotes the external force. If a harmonic force is seen as external force, then

(17) $$P(t)=P_{0}\sin(\Omega t)$$

where *P*_0_ is the amplitude of the moving harmonic load. The Ω is the excitation frequency of the moving harmonic load. 

The potential energy of the NES can be written as [29]:

(18) $$U^{N}=-\overset{L}{\underset{0}{\int }}\left[k_{N}{\left(y-w(x_{N},t)\right)}^{3}+c_{N}(\dot{y}-\ddot{w}(x_{N},t))\right]\delta (x-x_{N})w(x,t)dx$$

where *δ*(·) is the Dirac delta function, *x_N_* is the additional location of the NES, *y* is the displacement of the NES, and *k_N_*, *c_N_*, and, *m_N_* are the cubic stiffness, damping, and mass of the NES, respectively.

(19) $$U^{\prime }=U^{T}+U^{P}+U^{N}$$

Substituting Equations (7) and (9) into Equations (13) and (14), the strain and kinetic energies of the AFG beam based on CBT can be expressed as

(20) $$\begin{array}{l} U=\frac{1}{2}\int_{0}^{L}E(x)I(\frac{\partial^{2}w(x,t)}{\partial x^{2}}{)}^{2}dx\\ V=\frac{1}{2}\int_{0}^{L}\rho (x)A(\frac{\partial w(x,t)}{\partial t}{)}^{2}dx+\frac{1}{2}\int_{0}^{L}\rho (x)I(\frac{\partial^{2}w(x,t)}{\partial x\partial t}{)}^{2}dx \end{array}$$

where *I* means the second moment of the area, *ρ*(*x*) is the mass density, and *A* is the cross-section area, *A* = *bh*.

The governing equation in this study was approximated using the Ritz method, and the solution is assumed to take the following form:

(21) $$w(x,t)=\alpha^{T}a$$

where *α*(*x*) is a vector composed of an admissible function of the beam displacement and is expressed as $\alpha \left(x\right)={\left[\begin{array}{cccc} \alpha_{1}(x) & \alpha_{2}(x) & \cdots & \alpha_{m}(x) \end{array}\right]}^{T}$. *a*(*t*) is a vector composed of unknown generalized coordinates and is shown as $a\left(t\right)={\left[\begin{array}{cccc} a_{1}(t) & a_{2}(t) & \cdots & a_{m}(t) \end{array}\right]}^{T}$.

The type of boundary conditions plays a crucial role in determining the appropriate choice of admissible functions. In this study, the polynomial Ritz approximation function is selected as the admissible function for the analysis.

The polynomial admissible function for the AFG beam can be expressed as:

(22) $$\alpha_{i}(x)={\left(\frac{x}{L}\right)}^{l}{\left(1-\frac{x}{L}\right)}^{r}{\left(\frac{x}{L}\right)}^{i-1}$$

The indices of the admissible function for the different boundary conditions of the AFG beam were *l* and *r* at the left (*x* = 0) and right ends (*x* = *L*), respectively. The values of each index for different boundary conditions are listed in Table 1.

For the AFG beam, the Lagrangian equations are applied as follows [14]:

(23) $$\frac{d}{dt}(\frac{\partial V}{\partial {\dot{a}}_{i}})+\frac{\partial U}{\partial a_{i}}+\frac{\partial U^{\prime }}{\partial a_{i}}=0$$

where $a_{i}$ represents the unknown coefficients, and the overdot denotes the partial derivative with respect to time. The equation of motion for the NES is:

(24) $$m_{N}\ddot{y}+k_{N}{\left(y-w(x_{N},t)\right)}^{3}+c_{N}(\dot{y}-\dot{w}(x_{N},t))=0$$

Using Equations (19)–(21) and performing mathematical operations, Equation (23) yields the following form

(25) $$\rho (x)A\frac{\partial^{2}w}{\partial t^{2}}+\rho (x)I\frac{\partial^{4}w}{\partial x^{2}\partial t^{2}}-N_{x}^{Τ}\frac{\partial^{2}w}{\partial x^{2}}+E(x)I\frac{\partial^{4}w}{\partial x^{4}}=P(t)\delta (x-v_{p}t)$$

**Figure 3:**
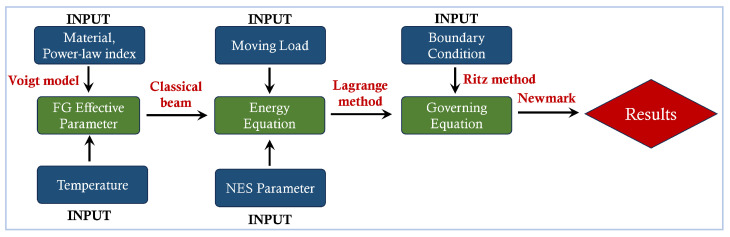
Flow chart for the derivation procedure.

By coupling Equations (24) and (25), the equation of motion for the entire system is derived. The dynamic response of the beam under the moving load is then solved using the Newmark method, with the Newton–Raphson method employed for iterative solution. To enhance clarity in understanding the derivation of the governing equation, a flowchart is provided in Figure 3.

## 3. Validation Studies

**Figure 4:**
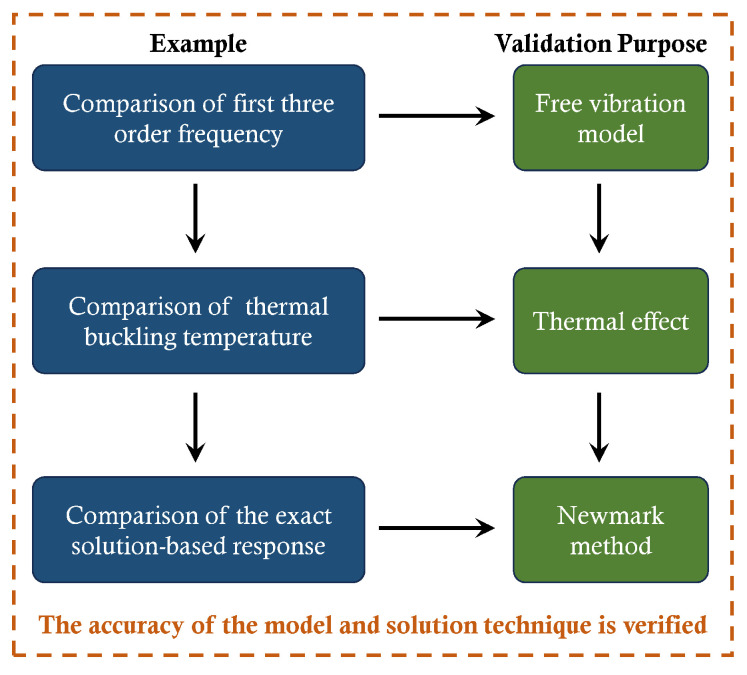
Flow chart for validation studies.

The accuracy of the established AFG structure and codes was verified by comparing our results with the published ones. To begin the validation section, a flow chart is provided in Figure 4, outlining the purpose of each validation example.

The ceramic was composed of silicon nitride, whereas the metal was composed of stainless steel. The left and right ends of the beam were ceramic-rich and metal-rich, respectively. The beam length, *L*, was 15.0 m and the height, *h*, and width, *b*, were both 1.0 m. The temperature-dependent material properties, *ξ*, of the ceramic and metal can be expressed as:

(26) $$\xi =\xi_{0}(\frac{\xi_{-1}}{T}+1+\xi_{1}T+\xi_{2}T^{2}+\xi_{3}T^{3})$$

where *ξ_i_* (*i* = 0, 1, 2, and 3) denotes the fitting coefficients obtained from experiments. Since the temperature dependence of the material was not considered here, the material coefficients used were those at ordinary temperatures, and the values of the individual coefficients are given in [30].

Table 2 lists the first three natural frequencies of the AFG beam based on CBT. The Voigt model was used for the calculations and temperature dependence was considered. The results agree well with the published ones.

Table 3 lists the critical buckling temperature of the AFG beam calculated using the CBT. The Voigt model was used for the calculation and temperature independence was considered. A good agreement was observed with the published values.

**Figure 5:**
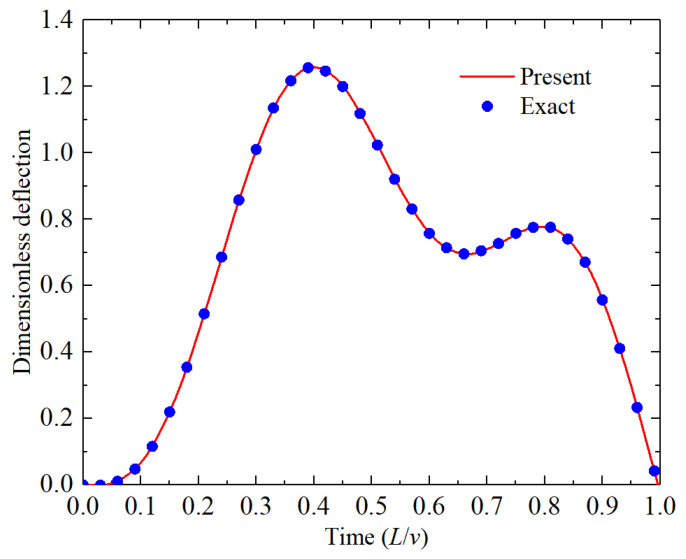
Comparison of the dynamic response of an isotropic beam subjected to a moving load.

In the last example, the dynamic response of an isotropic metal beam subjected to a moving load were studied and its results were compared with those from the exact solution [32]. Table 4 lists the material properties of aluminum. The geometric parameters of the beam were *L* = 20.0 m, *b* = 0.4 m, *H* = 0.9 m, and the speed of the moving load was 100 m/s. The load was set to be 48*E_m_*I/*L*^3^, and the dimensionless dynamic deflection was set to be *wL*^3^/48*E_m_*I. Figure 5 shows that the calculated results agree well with the results from the exact solutions.

## 4. Results and Discussion

### 4.1. Dynamic Response of AFG Beam with NES

In this section, the dynamic responses of AFG beams in thermal environments with an NES are presented. A ceramic–metal AFG beam was used for the calculations, with a length of 2.0 m, height of 0.05 m, and width of 0.02 m. The NES parameters were set as follows: mass *m_NES_* = 0.1 kg, stiffness *k*_NES_ = 1 × 10^5^ N/m^3^, and damping coefficient *c*_NES_ = 100 N·s/m. The beam was assumed to have clamped–clamped (C–C) boundary conditions. The ceramic and metal materials used were silicon nitride (Si_3_N_4_) and stainless steel (SUS304), respectively. The factor *k* was set to 5, and unless otherwise specified, this value was used in the parameter studies. The magnitude of the moving load was 1000 N, and the harmonic force had an excitation frequency of 300 Hz.

Firstly, the critical buckling temperatures and the first four natural frequencies of AFG beams are presented in Table 5 and Table 6, respectively, as these results serve as reference values for designing the parameters of the moving load. The numerical results indicate that, as the power-law index increases, both the critical buckling temperatures and natural frequencies decrease. This is attributed to the significant reduction in the percentage of Si_3_N_4_, which has a high Young’s modulus, as the power-law index increases.

**Figure 6:**
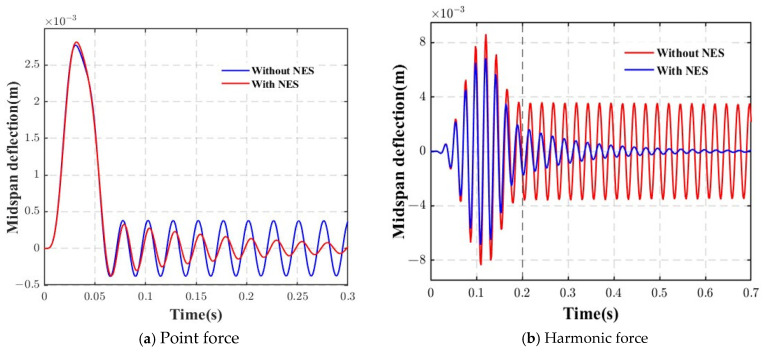
Time variation of the dynamic displacements at the midspan of the AFG beam at various loading velocities with or without NES.

Figure 6 depicts the responses of the deflections at the midspan of the AFG beam under moving loads at Δ*T* = 100 K. The velocity of the load was 30 m/s. The dynamic responses of the beams with and without NES were compared. From this figure, it can be seen that the NES effectively reduces the maximum deflection. When the load is still applied to the beam, the impact of the NES on the response is less significant, but it becomes more pronounced once the load leaves the beam, marking the onset of free vibration. Furthermore, for different types of loads, the NES proves to be more effective in suppressing the response generated by the moving harmonic load.

**Figure 7:**
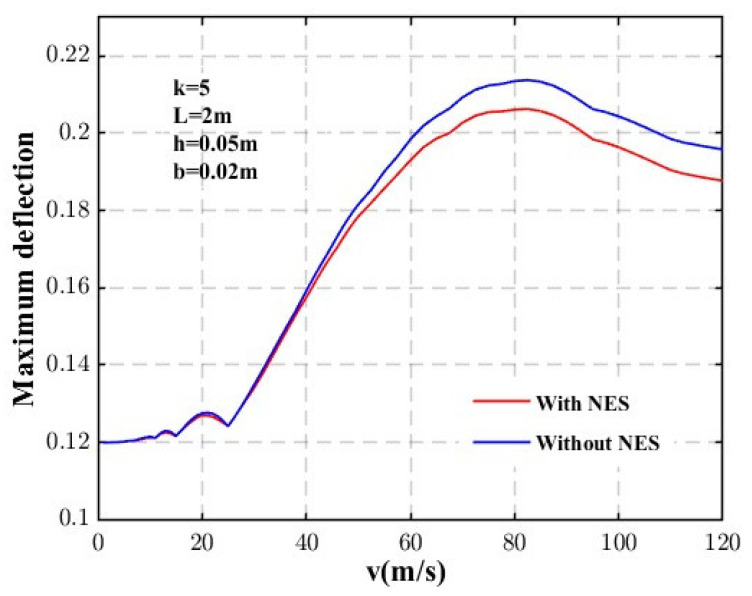
Maximum midspan deflection as a function of velocity.

The curves of the beam’s maximum deflection as a function of velocity are shown in Figure 7, comparing the effects with and without the NES. It can be observed that the midspan deflection initially increases with velocity until reaching a certain threshold, known as the critical velocity, after which it starts to decrease with further increases in velocity. This result demonstrates that the NES effectively reduces the maximum deflection at critical velocity, and its suppressive effect becomes more pronounced as the velocity increases.

**Figure 8:**
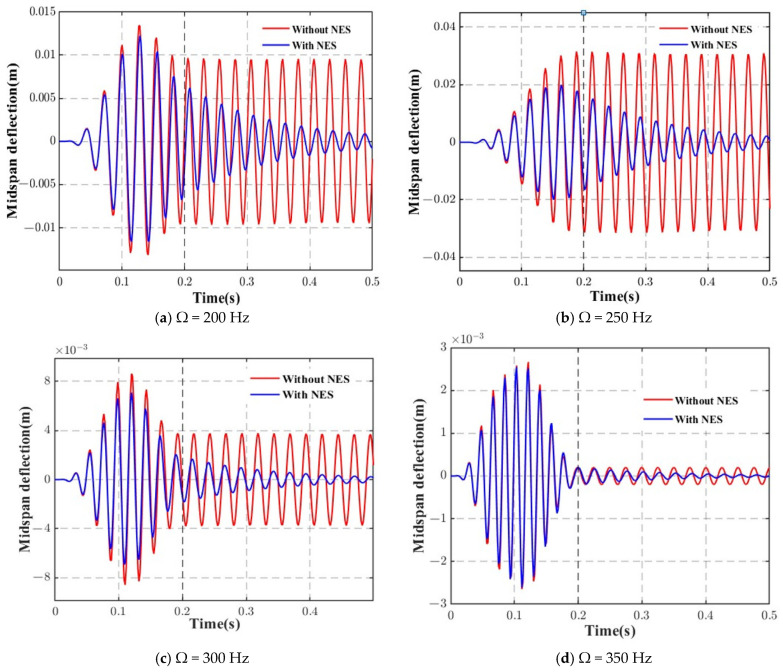
Time variation of the dynamic displacements at the midspan of the AFG beam at various load frequencies (harmonic force).

Figure 8 presents the response curves of the deflections at the midspan of the AFG beam under moving loads with different harmonic load frequencies at a constant velocity (*v* = 10 m/s), comparing the effects with and without the NES. The results show that the suppression effect of the NES varies with the load frequency. Among the four subfigures, the most obvious suppression effect can be observed when the load frequency is 250 Hz, which is near the fundamental frequency of the beam structure. From another perspective, it can be considered that optimizing the NES parameters allows for the best suppression effect on the structural response.

**Figure 9:**
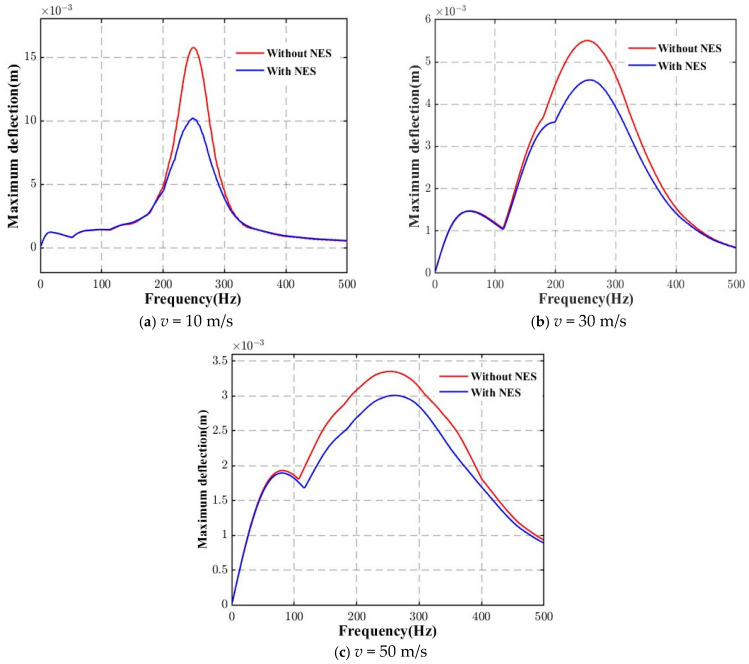
Relationship between the maximum midspan displacement and load frequency for a moving harmonic load.

The conclusion drawn above is further corroborated by the data presented in Figure 9, which demonstrates the relationship between the maximum midspan displacement and the load frequency. A notable peak is observed at a specific load frequency, which corresponds closely to the fundamental frequency of the AFG beam. For instance, a peak occurs at 250 Hz, which is in good agreement with the fundamental frequency of the AFG beam under thermal conditions (approximately 253 Hz when *k* = 5.0, as shown in Table 5). This resonance phenomenon occurs because the dynamic response of the beam is most sensitive at this frequency, which is characteristic of the natural vibration mode of the structure. As expected, the integration of the Nonlinear Energy Sink (NES) results in a significant reduction in the maximum deflection of the system. The suppression effect is most pronounced at the resonant frequency, where the NES effectively dissipates the vibrational energy, thereby mitigating the beam’s displacement. This reduction in amplitude validates the effectiveness of NES in vibration control, particularly in environments where thermal effects influence the mechanical properties of the AFG beam.

These findings not only confirm the capability of NES in reducing vibrations but also highlight its potential as a promising nonlinear absorber for enhancing the dynamic performance of AFG beams under thermal environments. The results emphasize the importance of optimizing NES parameters to achieve maximal vibration suppression and extend its applicability to a broader range of engineering problems involving complex dynamic environments.

### 4.2. Optimization of the NES Parameters

**Figure 10:**
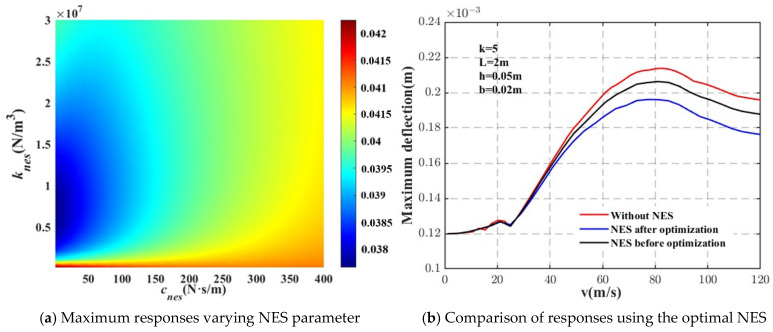
Effect of the NES optimization on the dynamic responses of the beam (point force).

**Figure 11:**
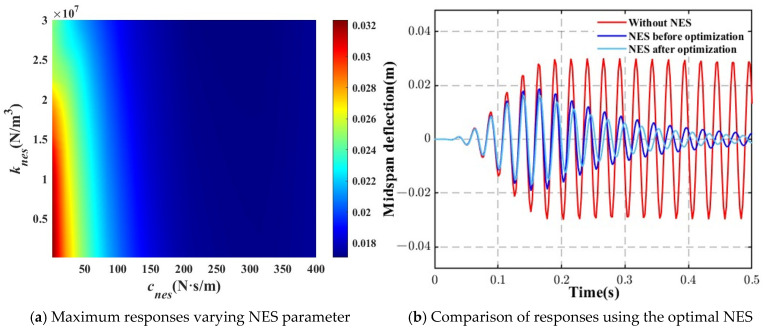
Effect of the NES optimization on the dynamic responses of the beam (harmonic force).

The NES parameters were optimized using the maximum deflection method, where the objective was to minimize the maximum deflection of the system by identifying the optimal set of NES parameters. The loads depicted in Figure 10 and Figure 11 represent point and harmonic forces, respectively. As observed in Figure 10, simply increasing the damping of the NES does not necessarily lead to an optimal design. Instead, both the stiffness and damping characteristics of the NES play critical roles. An appropriate balance between the damping and stiffness values is essential for achieving the most effective vibration control.

For the point force case, shown in Figure 10, the NES parameters were set as follows: *m_NES_* = 0.1 kg, *k_NES_* = 4 × 10^5^ N/m^3^, and *c_NES_* = 1 N·s/m. As shown in Figure 10b, with these optimized parameters, the NES significantly improved the vibration control of the beam. Specifically, at the critical speed, the maximum deflection of the system was reduced by 3.43% when the NES was not optimized. However, after optimization of the NES parameters, the maximum deflection was further reduced by 8.21%, resulting in a 2.4-fold increase in suppression efficiency.

Similarly, in the case of a harmonic force, as shown in Figure 11, the NES parameters were chosen as follows: *m_NES_* = 0.1 kg, *k_NES_* = 3 × 10^7^ N/m^3^, and *c_NES_* = 400 N·s/m. The results in Figure 11b further confirm that the optimized NES significantly enhances the vibration reduction, illustrating the effectiveness of parameter optimization in improving the system’s dynamic response.

This comprehensive analysis underscores the importance of optimizing both the damping and stiffness of the NES to achieve the most efficient vibration suppression across different loading scenarios.

## 5. Conclusions

In this study, a nonlinear damper, specifically the NES, was utilized to control the vibration of the AFG beam subjected to moving loads. The formulation was established using classical beam theory, and the equations of motion governing the system were derived via the Lagrange method. Numerical solutions were obtained using the Newmark method, assisted by the Newton–Raphson iteration technique. The NES parameters were optimized using the maximum deflection method. Parameter studies were conducted, and the findings not only confirmed the effectiveness of NES in reducing vibrations but also highlighted its potential as a promising nonlinear absorber for enhancing the dynamic performance of AFG beams in thermal environments. Key conclusions are summarized as follows:

1. The impact of NES on the dynamic response becomes more pronounced when the load exits the beam, marking the onset of free vibration. NES is particularly effective in suppressing the response induced by the moving harmonic load.
2. The NES significantly reduces the maximum deflection at critical velocity, with its suppression effect becoming more pronounced as velocity increases.
3. Achieving an optimal balance between damping and stiffness through NES parameter optimization is crucial for effective vibration control. In this study, a 2.4-fold increase in suppression efficiency was achieved.
4. The results underscore the importance of optimizing NES parameters to maximize vibration suppression and extend its applicability to a broader range of engineering problems involving complex dynamic environments.

An outlook on the present work is provided below:

1. The optimization was conducted solely based on the maximum deflection method, as it is the most direct approach for identifying the optimal NES parameters. However, alternative optimization criteria, such as energy dissipation rate or frequency response, could also be considered and may lead to more efficient designs.
2. Due to the absence of a temperature-dependent NES model, extreme thermal environments were not considered in the current study. This remains a valuable area for future research, and we are committed to developing such a model.

## Figures and Tables

**Table 1:** Indices of the admissible function for different boundary conditions.

Beam Theory	Boundary Conditions	Left End (*x* = 0)		Right End (*x* = *L*)	
		*l*	*p*	*r*	*p*
CBT	C–C	2	/	2	/
	C–S	2	/	1	/
	S–S	1	/	1	/
	S–C	1	/	2	/

**Table 2:** Natural frequencies of the AFG beam under CBT and their comparison with those published in a previous study.

C–C	First Order		Second Order		Third Order	
	Ref. [31]	Present	Ref. [31]	Present	Ref. [31]	Present
Si_3_N_4_	7.1835	7.1835	11.8898	11.8898	16.5683	16.5684
k = 0.2	6.5501	6.5505	10.8245	10.8253	15.0691	15.0702
k = 0.5	6.0126	6.0126	9.9698	9.9699	13.9006	13.9009
k = 1.0	5.5615	5.5614	9.2575	9.2575	12.9332	12.9332
k = 2.0	5.1865	5.1865	8.6540	8.6539	12.1077	12.1077
k = 5.0	4.9087	4.9087	8.1583	8.1583	11.4048	11.4052
k = 10.0	4.8318	4.8318	7.9848	7.9848	11.1368	11.1369
SUS304	4.7247	4.7247	7.8201	7.8201	10.8971	10.8972

**Table 3:** Critical buckling temperatures of the AFG beam under CBT and their comparison with those published in a previous study (unit: K).

C–C	Ref. [32]	Present	Error
Si_3_N_4_	1956.2	1957.33	0.058%
k = 0.2	1663.7	1664.73	0.062%
k = 0.5	1444.6	1445.03	0.030%
k = 1.0	1283.3	1283.68	0.030%
k = 2.0	1155.6	1156.68	0.093%
k = 5.0	1051.0	1052.99	0.189%
SUS304	954.4	954.91	0.053%

**Table 4:** Material properties of aluminum.

Material	*E*_m_ (GPa)	ν_m_	ρ_m_ (kg/m^3^)	*α*_m_ (1/K)
Alumina	390	0.30	3960	7.4764 × 10^−6^

**Table 5:** Critical buckling temperatures of the AFG beam.

C–C	Critical Buckling Temperatures
Si_3_N_4_	275.25
*k* = 0.20	234.10
*k* = 0.50	203.20
*k* = 1.00	180.52
*k* = 2.00	162.66
*k* = 5.00	148.08
SUS304	134.28

**Table 6:** First four natural frequencies of the AFG beam at Δ*T* = 100 K.

C–C	First Order	Second Order	Second Order	Fourth Order
Si_3_N_4_	755.25	2356.30	5132.92	9075.62
*k* = 0.1	663.70	2100.09	4542.86	8096.52
*k* = 0.5	473.65	1597.99	3473.43	6602.16
*k* = 1.0	380.39	1351.66	3000.72	5889.99
*k* = 5.0	253.49	1002.66	2387.88	4584.26
*k* = 10.0	236.09	949.78	2276.92	4260.89
SUS304	208.32	898.57	2113.95	3822.81

## Data Availability

The original contributions presented in this study are included in the article. Further inquiries can be directed to the corresponding author.

## Abbreviations

The following abbreviations are used in this manuscript:

AFG	Axially functionally graded
C–C	Clamped–Clamped
FG	Functionally graded
FGM	Functionally graded material
NES	Nonlinear energy sink
